# Is platelet-rich plasma an ideal biomaterial for arthroscopic rotator cuff repair? A systematic review and meta-analysis of randomized controlled trials

**DOI:** 10.1186/s13018-019-1207-9

**Published:** 2019-06-20

**Authors:** Changxu Han, Yuyan Na, Yong Zhu, Lingyue Kong, Tu Eerdun, Xuejun Yang, Yizhong Ren

**Affiliations:** 1 Department of Arthroscopy and Sports Medicine, The Second Affiliated Hospital of Inner Mongolia Medical University, No. 1 Yingfang Street, Huimin District, Hohhot, 010000 Inner Mongolia Autonomous Region China; 2 Department of Spinal Surgery, The Second Hospital of Inner Mongolia Medical University, No. 1 Yingfang Street, Huimin District, Hohhot, 010000 Inner Mongolia Autonomous Region China

**Keywords:** Platelet-rich plasma, Rotator cuff, Meta-analysis, Arthroscopic

## Abstract

**Background:**

Recently, many authors have reported the effects of platelet-rich plasma (PRP) on rotator cuff repair. Whether PRP treatment during arthroscopic rotator cuff repair improves tendon healing rates or restores full function remains unknown. The purpose of this meta-analysis was to evaluate the clinical improvement and radiological outcomes of PRP treatment in patients undergoing arthroscopic rotator cuff repair.

**Methods:**

PubMed, EMBASE, and the Cochrane Central Register of Controlled Trials were searched. The study included only level 1 or 2 randomized controlled trials (RCTs) that compared the injection of platelet-rich plasma or platelet-rich fibrin matrix. The methodological quality of the trials was assessed using the Cochrane Handbook for Systematic Reviews of Interventions, 5.3. Continuous variables were analysed using the weighted mean difference, and categorical variables were assessed using relative risks. *P* < 0.05 was considered statistically significant.

**Results:**

The meta-analysis revealed a lower retear rate following PRP treatment than that following the control method (mean difference, 1.10; 95% CI, 1.03 to 1.18; *P* = 0.004). Constant shoulder scores improved with PRP (mean difference, 2.31; 95% CI, 1.02 to 3.61; *P* = 0.0005). PRP treatment also resulted in higher UCLA scores (mean difference, 0.98; 95% CI, 0.27 to 1.69; *P* = 0.007), and simple shoulder test scores were improved (mean difference, 0.43; 95% CI, 0.11 to 0.75; *P* = 0.008). Finally, lower visual analogue scale scores were observed with PRP augmentation (mean difference, − 0.35; 95% CI, − 0.57 to − 0.13; *P* = 0.002).

**Conclusions:**

The current systematic review and meta-analysis reveals that PRP treatment with arthroscopic repair of rotator cuff tears decreases the retear rate and improves the clinical outcomes.

**Systematic review registration:**

PROSPERO CRD42016048416

## Introduction

The use of autologous platelet-rich plasma (PRP) or similar products containing platelets has been widely studied in bone and tendon tissue healing and reconstruction [1–5]. PRP is known to contain more than 1500 bioactive proteins that are important for tendon healing, including growth factors such as transforming growth factor beta (TGF-ß), fibroblast growth factor (FGF), and platelet-derived growth factor (PDGF) [6, 7].

PRP, glucocorticoids, local anaesthetics, or hyaluronic acid are used to reduce pain and improve performance in patients who undergo rotator cuff repair. Among them, local anaesthetics and glucocorticoids have cytotoxic effects on tenocytes, and hyaluronic acid decreases pain in patients with partial tear of the rotator cuff tendons [8]. Recently, many authors have reported the effects of PRP on partial or complete tears of rotator cuff tendons [9–11]. These trials returned mixed results [9–14] and were unable to show consistently improved retear rates or improved clinical outcome scores. Although some meta-analyses on this topic have been published [15–19], these have also returned mixed results. Whether PRP treatment during arthroscopic rotator cuff repair improves tendon healing rates or restores of full function remains unknown.

The aim of this study was to conduct a systematic review and meta-analysis of level I and level II studies to investigate the clinical and imaging outcomes of PRP treatment during arthroscopic repair of rotator cuff tears. Our hypothesis is that PRP application deceases retear rates and improves clinical outcomes.

### Literature search

We searched the Cochrane Central Register of Controlled Trials (2016 Issue 2), EMBASE (1980 to 2016 Week 36), and PubMed (1946 to September 2016). No language restrictions were applied. Search terms were as follows: platelet-rich plasma OR plasma OR platelet-rich OR platelet gel OR platelet plasma OR PRP OR PRFM OR platelet-rich fibrin matrix OR PRFM OR platelet AND rotator cuff OR supraspinatus tendon OR supraspinatus. The references of published studies were assessed by manual search to identify additional articles. Finally, we searched the following journal contents within the previous 5 years for randomized controlled trials: British Journal of Sports Medicine, the Journal of Shoulder and Elbow Surgery, Arthroscopy, The Journal of Arthroscopic and Related Surgery, and The American Journal of Sports Medicine.

### Eligibility criteria

Inclusion criteria were as follows: studies of patients diagnosed with rotator cuff tears requiring arthroscopic repair; level I or II randomized controlled trials; studies in which the treatment group received an injection of platelet-rich plasma or platelet-rich fibrin matrix; studies with patients aged 18 years or older; studies with adequate statistical power to defect differences with 95% confidence intervals (CIs); studies with a minimum of one of the following outcome measurements performed postoperatively: American Shoulder and Elbow Surgeons (ASES) score, constant shoulder score, University of California at Los Angeles (UCLA) score, Simple Shoulder Test score, with radiography (MRI and/or USG); studies with patient follow-up > 80%; studies with a minimum follow-up of 6 months; and studies with no restrictions on treatment dosage, usage of procedures, or number of injections.

The exclusion criteria were as follows: retrospective studies; case-control studies; case reports; studies without abstracts; level III or IV evidence studies; studies of patients with a history of previous injury or surgery to the same shoulder, with postoperative infection, with rheumatoid arthritis, or with arthrofibrosis; studies with inadequate follow-up; studies reporting outcomes only after PRP treatment; and studies including open or mini-open surgical procedures.

### Data extraction

Extraction of all variables and outcomes of interest and assessment of methodological quality were performed independently by two authors (C-X.H. and Y-Y.N.). Reviewers were not blinded to the study authors, journal, or source of financial support. Disagreements were resolved through discussion and, when necessary, by consultation with a third author (Y-Z.R.). The following data/information were extracted from the studies that met the inclusion criteria: first author’s name; publication year; percent of males; mean age; number of patients; population differences; repair type; PRP types; clinical and imaging follow-up intervals; clinical outcome scores; and the number of retears in each study group and control group.

### Assessment of methodological quality

The methodological quality of the trials was assessed using the Cochrane Handbook for Systematic Reviews of Interventions, 5.3. To determine the possibility of bias, we examined random sequence generation, allocation concealment, blinding of patients and personnel, blinding of outcome assessment, incomplete outcome data, and selective reporting risk. Risk of bias figures were generated using Cochrane Review Manager software 5.3.

### Assessment of heterogeneity

The heterogeneity of each study was assessed by two separate reviewers (C-X.H. and Y-Y.N.), based on the Cochrane Handbook for Systematic Reviews of Interventions [20]. Differences of opinion between reviewers were resolved by discussion and consultation with a third author (Y-Z.R.). Both clinical heterogeneity (e.g. differences among patients, interventions, and outcomes) and statistical heterogeneity (variation between trials in the underlying treatment effects being evaluated [21]) were considered. We assessed heterogeneity by visual inspection of the forest plots. To determine inconsistencies in the study results, statistical heterogeneity between studies was formally tested with a standard c-square test. We used the *I*^2^ test to provide an objective measurement of statistical heterogeneity. According to the Cochrane Handbook [22], heterogeneity was quantified using the *I*^2^ statistic with a rough guide for interpretation as follows: 0 to 40%—no heterogeneity, 30 to 60%—moderate heterogeneity, 50 to 90%—substantial heterogeneity, and 75 to 100%—considerable heterogeneity. A fixed effects model was used if the *I*^2^ values were less than 60%; otherwise, a random effects model was used. Tests for significance were two-tailed, and *P* < 0.05 was deemed significant.

### Subgroups and sensitivity

Subgroup analyses and sensitivity values were used to assess factors responsible for potential heterogeneity. We were unable to perform all planned analyses due to the lack of data (see differences between protocol and review). Analyses were dependent on the number of studies included and the availability of appropriate outcomes and covariates. We further investigated heterogeneity by observing the effects of removing single trial outliers. If there was heterogeneity across studies, studies were categorized into various subgroups (e.g. tear size).

We performed sensitivity analyses (the leave-one-out approach) to evaluate the impact of removing from the analysis studies at high or unclear risk of selection bias (primarily in terms of inadequate allocation concealment) and those with detection bias (lack of assessor blinding).

### Statistical analysis

Statistical analysis was performed using Review Manager 5.3 (Cochrane Collaboration, Nordic Cochrane Centre, Copenhagen, Denmark). Continuous variables were analysed using the weighted mean difference, and categorical variables were assessed using relative risks. *P* < 0.05 was considered statistically significant, and 95% CIs are reported. Homogeneity was tested by the *Q* statistic (significance level at *P* < 0.1) and the *I*^2^ statistic (significance level at *I*^2^ > 50%). A random effects model was used if the *Q* or *I*^2^ value was statistically significant; otherwise, a fixed effects model was used. In addition, only outcomes reported by four or more studies were pooled to ensure good validity and high quality. Fewer than four references created an excessive opportunity for bias [23]. If a study reported the preoperative baseline of an outcome (e.g. shoulder score) and it was not similar between the two groups, this outcome was not put into the pool in our meta-analysis.

## Results

**Fig. 1:**
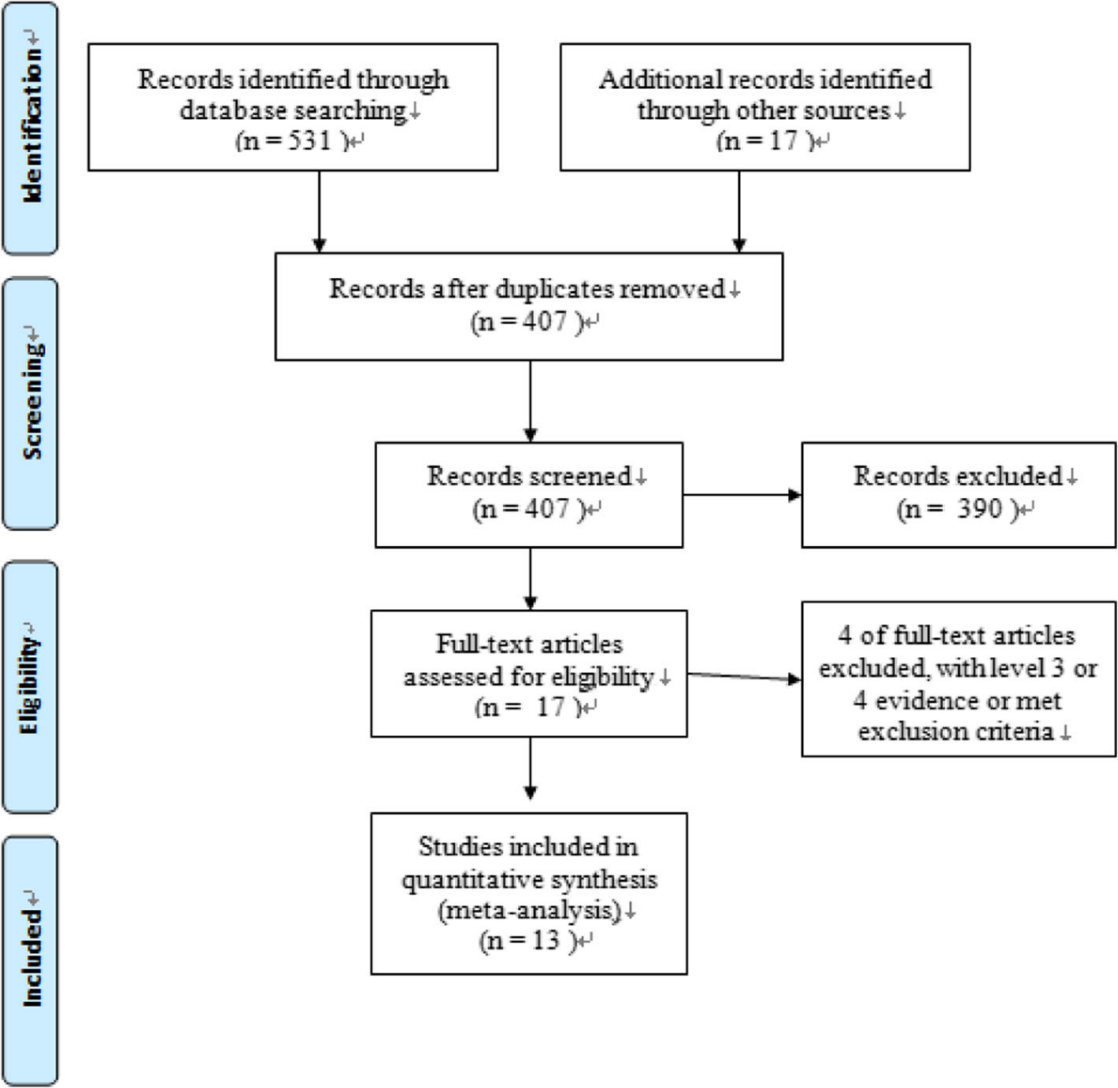
Flow diagram summarizing the process by which the 13 included studies were identified

A flow diagram outlining the process for study selection is shown in Fig. 1. A total of 513 potentially relevant articles were identified after duplicates were removed.

After screening titles and abstracts, 390 records were eliminated, leaving 14 studies for further review. Fourteen articles [9, 24–35] met eligibility criteria. Two studies [24, 34] were derived from the same randomized controlled trial. The follow-up times of these two studies were different (1 and 2 years). We extracted data from the article reporting the 1-year follow-up [34] to ensure a similar time of outcome assessment with other included studies.

### Study characteristics

The principal study characteristics are displayed in Table 1. A total of 880 patients (439 in the PRP application groups and 441 in the control groups) were included, with individual sample sizes ranging from 28 to 88 patients. The patient age range was 29 to 77 years. The gender distribution between the two groups was similar. The final follow-up was 6 to 16 months post-treatment. Table 2 displays the distinctive characteristics of each study, including tear type, rotator cuff repair techniques, method of PRP preparation, subjective outcomes, and relevant findings. In addition, the PRP type and injection characteristics of the included studies are listed in Table 3. Besides, PRP type and injection characteristics of the included studies are listed in Table 3.

**Table 1:** Characteristics of included randomized controlled trials. *PRP* platelet-rich plasma

Authors	Publish year	Male % (PRP+/PRP−)	Mean age (PRP+/PRP−)	Shoulders analysed (PRP+/PRP−)	Minimum imaging follow-up, months	Minimum clinical follow-up, months
Castricini et al. [25]	2011	40 (17/23)	(55.5/55.2)	88 (43/45)	16	16
Randelli et al. [31]	2011	21 (8/13)	(61.3/59.5)	45 (22/23)	12	12
Gumina et al. [27]	2012	41 (20/21)	61 (60/63)	76 (39/37)	12	12
Weber et al. [35]	2012	36 (20/16)	(59.7/64.5)	59 (29/30)	12	12
Jo et al. [28]	2013	24 (10/14)	(64.2/61.9)	47 (24/23)	9	12
Ruiz-Moneo et al. [33]	2013	25 (14/11)	(56/55)	63 (32/31)	12	12
Malavolta et al. [30]	2014	17 (8/9)	(55.3/54.1)	54 (27/27)	12	12
Sánchez Márquez et al. [34]	2011	8 (NR/NR)	65 (NR/NR)	28 (14/14)	12	12
Rodeo et al. [32]	2012	44 (23/21)	(58.9/57.2)	67 (35/32)	3	12
Flury et al. [26]	2016	38(18/20)	58.9 /57.8	103 (49/54)	24	24
Holtby et al. [9]	2016	41(20/21)	59/59	74 (36/38)	6	6
Pandey et al. [10]	2016	74(38/36)	54.8 /54.1	102 (52/50)	24(12)	24(12)
Jo et al. [29]	2015	17(8/9)	60.8/60.92	74(37/37)	12	12

**Table 2:** Summary of included randomized controlled trial

Authors/publish year	Population differences	Repair type	Outcomes measured	Relevant findings
Castricini et al. 2011 [25]	Included any full-thickness tear	Double row	Subjective: Constant scores Imaging: MRI at 16 months	No difference in constant scores and retear rates between groups
Randelli et al. 2011 [31]	Included any full-thickness tear	Single row	Subjective: Constant, UCLA, SST Imaging: MRI 12 months	Significant improvement in constant, UCLA, and SST in PRPþ group No difference in outcomes at final follow-up
Gumina et al. 2012 [27]	Included only large tears Excluded partial tears, massive tears, traumatic tears	Single row	Subjective: Constant, ST Imaging: MRI at 12 months	Significantly increased constant score in the PRPþ group, but no difference in change from pre- to postoperatively
Weber et al. 2012 [35]	Included any arthroscopic rotator cuff repair	Single row	Subjective: ASES, UCLA, SST, VAS Imaging: MRI at 12 months ROM	No difference in outcome scores or ROM between groups No difference in retear rates between groups
Jo et al. 2013 [28]	Included only large tears (> 3 cm sagittal length) Included 4 partial repairs	Double row	Subjective: ASES, CLA, Constant, SST, DASH, SPADI Imaging: MRI or CTA at 9 months	No difference between the two groups on the VAS for pain, ROM, muscle strength, overall satisfaction, and function The retear rate of the PRP group was significantly lower
Ruiz-Moneo et al. 2013 [33]	Included tendon retraction and fatty infiltration, smokers	Double row	Subjective: UCLA Imaging: MRA at 12 months	No difference in UCLA scores between groups No difference in retear rates between groups
Malavolta et al. 2014 [30]	Included only tears < 3 cm in sagittal length	Single row	Subjective: Constant, UCLA Imaging: MRI at 3, 6, and 12 months	No differences in constant or UCLA scores between groups No difference in retear rates between groups
Sánchez Márquez et al. 2011 [34]	Included only repairable large tears > 5 Excluded subscapularis tears	Single row	Subjective: Constant Imaging: MRA at 12 months	No differences in constant or UCLA scores between groups No difference in retear rates between groups
Rodeo et al. 2012 [32]	Included full-thickness tears, age > 40 years	Double row	Subjective: ASES, L’Insalata Imaging: US at 12 weeks	No difference in outcome scores between groups No difference in retear rates between groups
Flury et al. 2016 [26]	A complete rotator cuff tear	Double row	Subjective: Constant-Murley score, ASES, OSS Imaging: MRI or US at 12 months	No significantly improved function at 3, 6, and 24 months after arthroscopic repair compared with control patients receiving ropivacaine
Holtby et al. 2016 [9]	Full-thickness and partial-thickness tear	Single row and double row	Subjective: VAS, CMS, ASES, ShortWORC Imaging: MRI at 6 months	A short-term effect on perioperative pain No significant impact on patient-oriented outcome measures or retear rate
Pandey et al. 2016 [10]	Medium-sized to large cuff tears	Single row	Subjective: VAS, CMS, ASES, UCLA Imaging: US at 24 months	Retear in the PRP group was significantly lower, significant improvement in constant, UCLA score No difference in ASES score
Jo et al. 2015 [29]	Medium to large rotator cuff tears	Double row	Subjective: Constant score, VAS, ASES, UCLA, SST, SPADI scores Imaging: MRI at 12 months	A decreased retear rate of the supraspinatus, but not the speed of healing No significantly improved function scores at and 12 months after arthroscopic

**Table 3:** PRP type and injection characteristics

Study	Leukocyte-poor/rich PRP	Volume (ml)	Activating agent	Applied site
Castricini et al. 2011 [25]	Leukocyte-rich PRP	NR	Not report	Bone-tendon interface
Randelli et al. 2011 [31]	Leukocyte-rich PRP	6	Calcium chloride	Bone-tendon interface and subacromial space
Gumina et al. 2012 [27]	Leukocyte-rich PRP	5.2	Calcium gluconate	Bone-tendon interface
Weber et al. 2012 [35]	Leukocyte-poor PRP	1	Calcium	Bone-tendon interface
Jo et al. 2013 [28]	Leukocyte-poor PRP	9	Calcium gluconate	Bone-tendon interface
Ruiz-Moneo et al. 2013 [33]	Leukocyte-poor PRP	1	Calcium chloride	Bone-tendon interface
Malavolta et al. 2014 [30]	Leukocyte-poor PRP	10	Calcium chloride	Bone-tendon interface
Sánchez Márquez et al. 2011 [34]	Leukocyte-poor PRP	7	Not report	Bone-tendon interface
Rodeo et al. 2012 [32]	Leukocyte-poor PRP	9	Calcium chloride	Bone-tendon interface
Flury et al. 2016 [26]	Leukocyte-poor PRP	4	Not report	Bone-tendon interface
Holtby et al. 2016 [9]	Leukocyte-poor PRP	7	Not report	Bone-tendon interface
Pandey et al. 2016 [10]	Leukocyte-poor PRP	8	Calcium chloride	Bone-tendon interface
Jo et al. 2015 [29]	Leukocyte-poor PRP	9	Calcium gluconate	Bone-tendon interface

### Risk of bias assessment of the randomized controlled trials (RCTs)

**Fig. 2:**
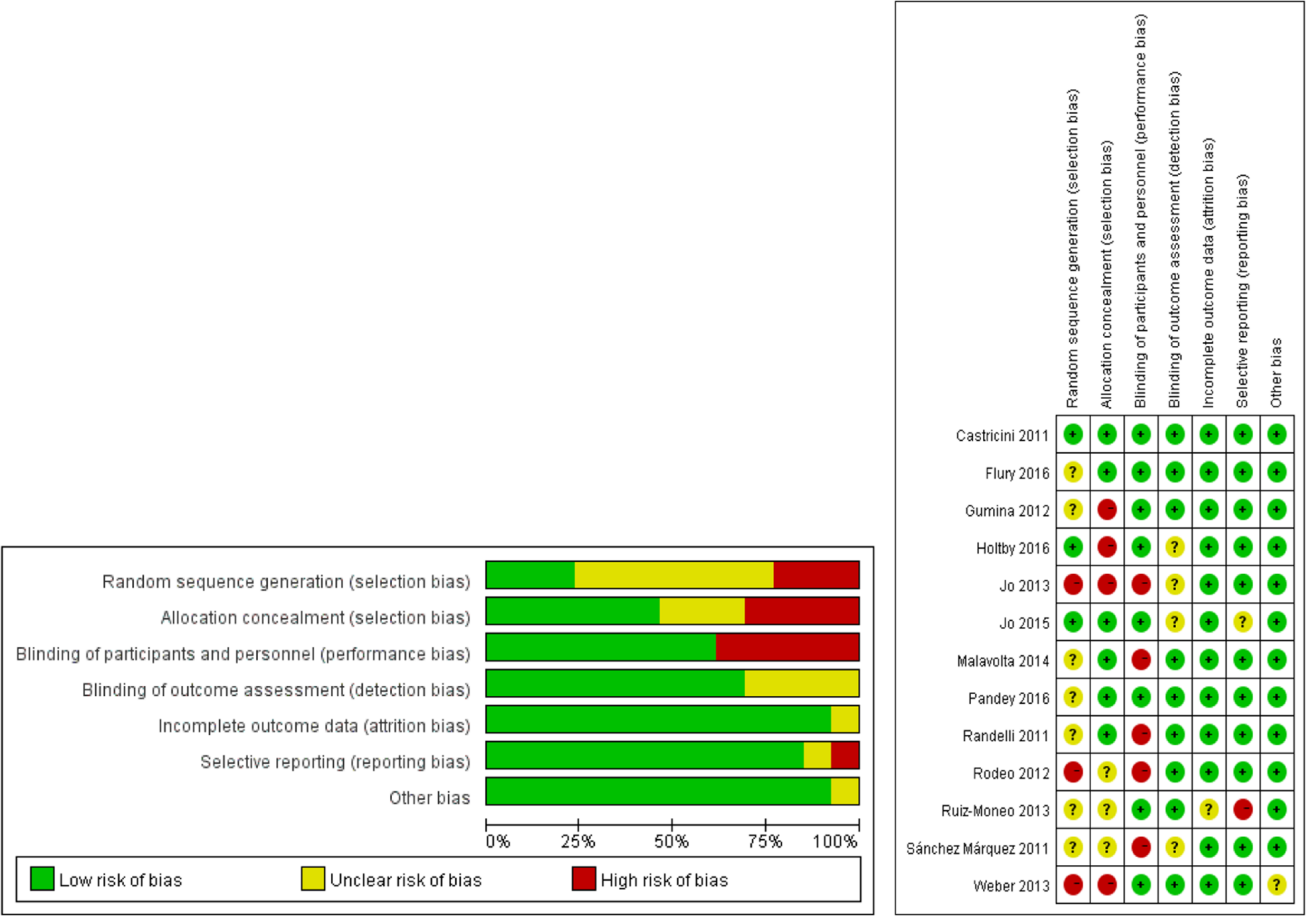
Risk of bias summary and graph of the included studies

All included studies reported the level of evidence in the publication itself (therapeutic level I in 11 studies [9, 10, 25–31, 33, 35] and therapeutic level II in two studies [32, 34]). The risk of bias assessed by the Cochrane Collaboration’s tool for qualitative parts is shown in Fig. 2. The studies had a low to medium risk of bias. A risk of bias was found in four of 13 studies (30.2%) due to randomization procedures (allocation concealment bias [9, 27, 28, 35]) and in five of 13 (38.5%) studies [28, 30–32, 34] related to performance bias. In seven of 13 studies (45.5%), the completeness of randomization procedures (selection bias) was unclear, either because of the absence of a Consolidated Standards of Reporting Trials statement or because of the absence of an intention-to-treat analysis [10, 26, 27, 30, 31, 33, 34].

### Retear rate

**Fig. 3:**
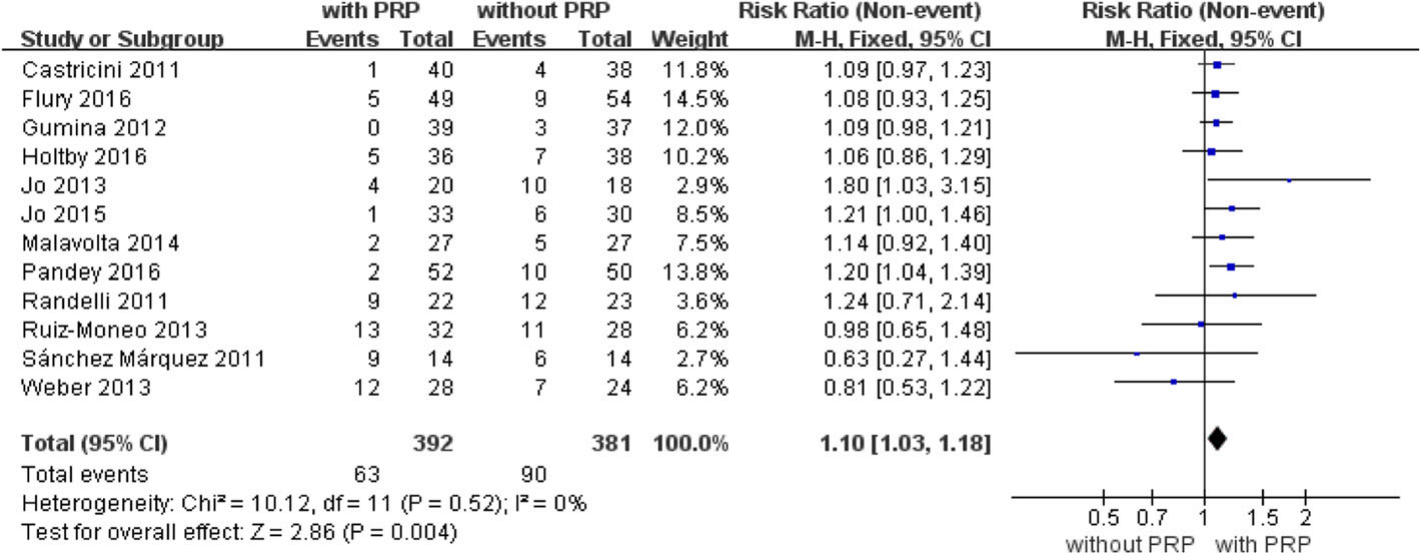
Forest plot for retear rate. A fixed-effects model was used because of the acceptable heterogeneity (I2= 0%). The size of each square is proportional to the weight of the study. The dark diamond on the right of the vertical line, indicating that the retear rate was lower after PRP application than control groups. (CI, confidence interval; df, degrees of freedom; I2, heterogeneity test; M-H, Mantel-Haenszel; PRP, platelet-rich plasma; z, P value of weighted test for overall effect)

Twelve randomized controlled trials with a total of 773 patients reported a retear rate at the last follow-up ([9, 10, 25–31, 33–35], Fig. 3). Retears occurred in 63 (16%) of 392 patients in the platelet-rich plasma group and in 90 (24%) of 381 patients in the control group (mean difference, 1.10, 95% CI, 1.03 to 1.18, *P* = 0.004, Fig. 3). The integrity of the repaired rotator cuff was evaluated by magnetic resonance imaging (MRI) in several studies [9, 25, 27, 29–31, 33–35]. MRI or computed tomographic arthrography was reported in one study [28], MRI or ultrasonography in one study [26], and ultrasonography in one study [10]. The test for heterogeneity showed no significant heterogeneity of the pooled results (*I*^2^ = 0%; *P* = 0.52). No further analysis was possible.

### Constant score

**Fig. 4:**
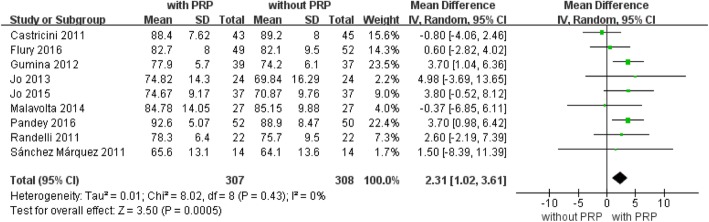
Forest plot for Constant shoulder score. A fixed-effects model was used because of no heterogeneity (I2 = 0%). The size of each square is proportional to the weight of the study. The dark diamond on the right of the vertical line, indicating that the Constant Score was higher after PRP application than control groups. (CI, confidence interval; df, degrees of freedom; I2, heterogeneity test; IV, inverse variance; PRP, platelet-rich plasma; SD, standard deviation; z, P value of weighted test for overall effect)

Constant shoulder scores at the last follow-up were reported for 615 patients in nine studies [10, 25–31, 34], Fig. 4). Significant differences were found in the fixed effects model between the PRP+ and PRP− treatment groups at the last follow-up along their respective recovery paths (*P* = 0.0005). These data suggest that constant shoulder score improvement may be accelerated by PRP treatment in arthroscopic repair of rotator cuff tears (mean difference, 2.31; 95% CI, 1.02 to 3.61; *P* = 0.0005, Fig. 4). No statistical heterogeneity was found (*I*^2^ = 0%; *P* = 0.43). No further analysis was possible.

### University of California at Los Angeles (UCLA) score

**Fig. 5:**
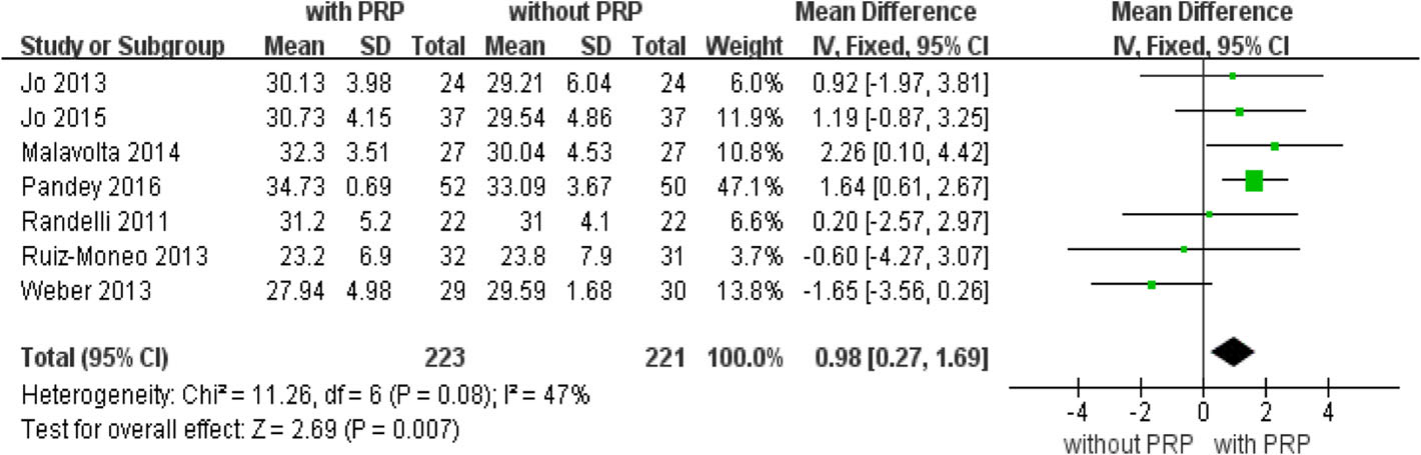
Forest plot for University of California at Los Angeles (UCLA) shoulder score. A fixed-effects model was used because of no heterogeneity (I2= 0%). The size of each square is proportional to the weight of the study. The dark diamond on the right of the vertical line, indicating that UCLA was higher after PRP application than control groups. (CI, confidence interval; df, degrees of freedom; I2, heterogeneity test; IV, inverse variance; PRP, platelet-rich plasma; SD, standard deviation; z, P value of weighted test for overall effect)

Seven trials [10, 28–31, 33, 35] with a total of 444 patients reported UCLA score outcomes at the end of follow-up (Fig. 5). The pooled data in the fixed effects analysis showed a significantly higher UCLA score with PRP treatment (mean difference, 0.98; 95% CI, 0.27 to 1.69; *P* = 0.007, Fig. 5). Heterogeneity across the studies was moderate (*P* = 0.08; *I*^2^ = 47%).

### American Shoulder and Elbow Surgeons (ASES) score

**Fig. 6:**
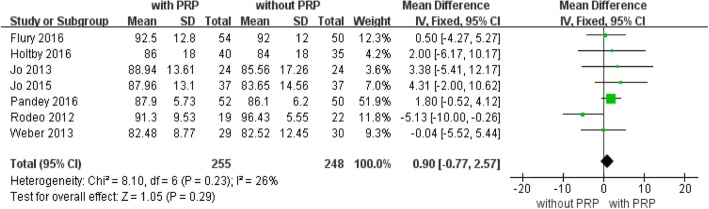
American Shoulder and Elbow Surgeons (ASES). A fixed-effects model was used because of no heterogeneity (I2 = 26%). The dark diamond intersects the vertical line, indicating that ASES was higher after PRP application than control groups. (CI, confidence interval; df, degrees of freedom; I2, heterogeneity test; IV, inverse variance; PRP, platelet-rich plasma; SD, standard deviation; z, P value of weighted test for overall effect)

Seven studies with a total of 503 patients available at the latest follow-up reported ASES scores (Fig. 6). Fixed-effects analysis showed that the difference was not significant between the two groups (mean difference, 0.90; 95% CI, − 0.77 to 2.57; *P* = 0.23, Fig. 6). No significant heterogeneity was found (*I*^2^ = 26%; *P* = 0.23). No further analysis was possible.

### Simple Shoulder Test (SST) score

**Fig. 7:**
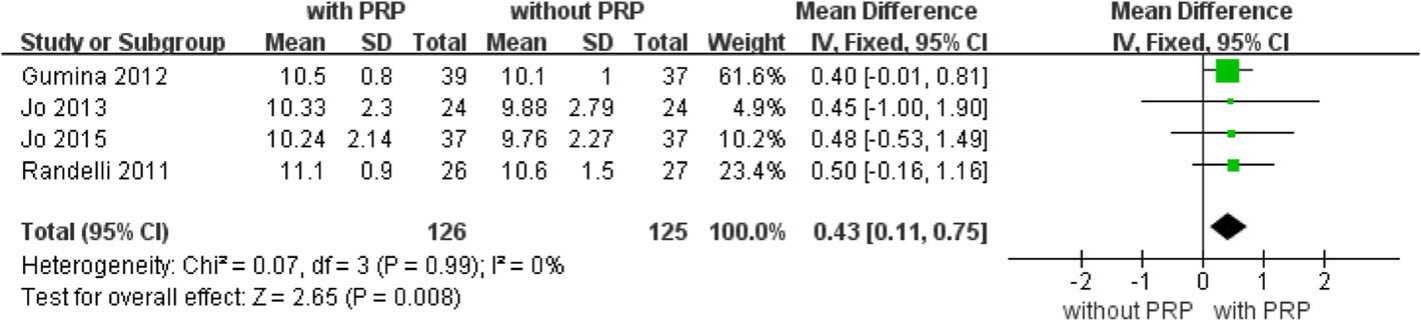
Forest plot of Forest plot of Simple Shoulder Test (SST) score. A fixed-effects model was used because of no heterogeneity (I2= 0%). The size of each square is proportional to the weight of the study. The dark diamond on the right of the vertical line, indicating that SST was higher after PRP application than control groups. (CI, confidence interval; df, degrees of freedom; I2, heterogeneity test; IV, inverse variance; PRP, platelet-rich plasma; SD, standard deviation; z, P value of weighted test for overall effect)

Four studies [27–29, 31] with a total of 251 patients available at the latest follow-up reported data on SST scores (Fig. 7). The forest plot showed significantly higher SST scores with PRP augmentation (mean difference, 0.43; 95% CI, 0.11 to 0.75; *P* = 0.008, Fig. 7). No statistical heterogeneity was found (*I*^2^ = 0%; *P* = 0.99). No further analysis was possible.

### Visual analogue scale (VAS) pain scores

**Fig. 8:**
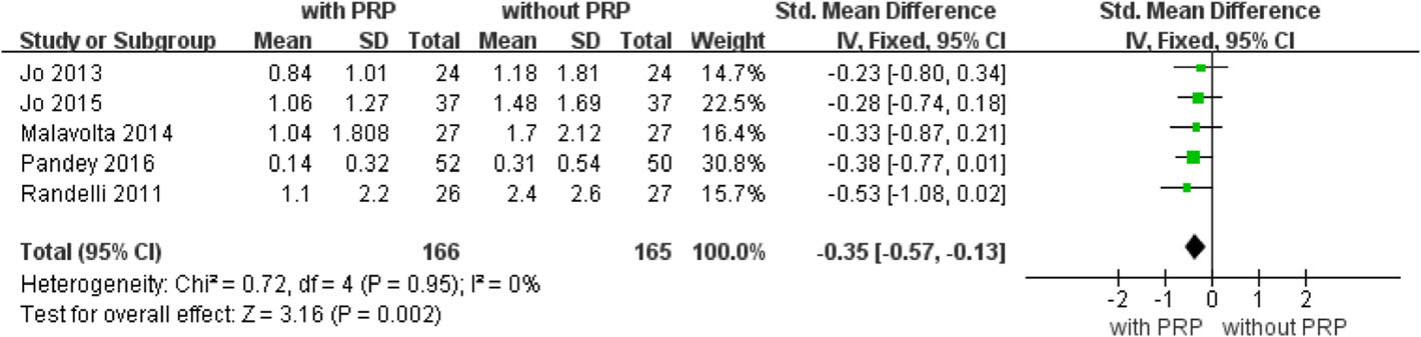
Forest plot of Forest plot of Visual Analogue Scale (VAS) pain score. A fixed-effects model was used because of no heterogeneity (I2= 0%). The size of each square is proportional to the weight of the study. The dark diamond on the left of the vertical line, indicating that indicating that VAS was higher after PRP application than control groups.(CI, confidence interval; df, degrees of freedom; I2, heterogeneity test; IV, inverse variance; PRP, platelet-rich plasma; SD, standard deviation; z, P value of weighted test for overall effect)

Five of the 13 studies [10, 28–31] provided complete data regarding visual analogue scale pain scores at pre- and post-treatment. Five studies with a total of 331 patients with available data at the latest follow-up reported VAS scores (Fig. 8). The forest plot showed significantly lower VAS scores with PRP treatment (mean difference, − 0.35; 95% CI, − 0.57 to − 0.13; *P* = 0.002). No statistical heterogeneity was found (*I*^2^ = 0%; *P* = 0.95). No further analysis was possible.

## Discussion

Rotator cuff tears occur as a result of normal ageing, excessive loading, and microtrauma. They are common in the general population and can have serious effects on a person’s work and life [36–38]. Several therapies have been reported; however, the problem can be difficult to manage. Thus, attention has turned to novel treatments [37, 39]. PRP has been investigated for its biological effects on the human rotator cuff [40, 41]. However, the available evidence to support treatment is inadequate and even conflicting. Thus, we conducted this meta-analysis of 13 RCTs to compare the efficacy of platelet-rich plasma or platelet-rich fibrin matrix application in conjunction with arthroscopic rotator cuff repair.

This meta-analysis of level I and level II studies examined the efficacy of PRP therapy in arthroscopic rotator cuff repairs. The main findings of the current study were that the use of PRP in rotator cuff repair had a significantly positive effect on postoperative retear rates and on functional outcome measures, including constant shoulder scores, constant pain scores, UCLA shoulder scores, and VAS scores. These results supported our primary hypothesis that platelet-rich plasma deceases retear rates and improves functional outcomes following arthroscopic rotator cuff repair.

As a potential biological product, PRP has been widely used to promote the healing of bones, cartilage, and tendons [42–47]. PRP is rich in soluble growth factors that may be involved in tissue regeneration [48, 49]. When these growth factors are released from platelets, they trigger tissue regeneration [50–52]. Some animal studies have shown beneficial effects on the initial stage of rotator cuff tendon-to-bone healing following PRP treatment [53–55]. Hapa et al. [55] found that local autologous platelet-rich plasma injection may have beneficial effects on initial rotator cuff tendon-to-bone healing and may enhance initial tendon-to-bone healing remodelling in vivo. Beck et al. [53] reported that PRP and platelet-rich fibrin matrix significantly improved tendon-to-bone healing of repaired rat supraspinatus tears. In addition, PRP is being investigated for its biological effects on the human rotator cuff. Randelli et al. [11] first reported an uncontrolled pilot study of arthroscopic rotator cuff repair with PRP leading to improved pain and functional outcomes without any adverse events. Pandey et al. [10] found superior structural healing of arthroscopic repair of the large rotator cuff tears when treated with moderately concentrated PRP. PRP also accelerated the vascularity of the rotator cuff and surrounding tissues in the early healing phase. However, Holtby et al. [9] reported a prospective, double-blinded randomized controlled trial of arthroscopic rotator cuff repair with PRP showing improved short-term effects on perioperative pain without any significant impact on patient-oriented outcome measures or on structural integrity of the repair.

Although several meta-analyses evaluated the outcomes of arthroscopic rotator cuff surgery with PRP, these studies returned mixed results [16–19, 56, 57]. The routine use of PRP for arthroscopic rotator cuff repair is not warranted on the basis of these meta-analyses, as they have been unable to show any overall clinical superiority versus the control repair regimen. Whether PRP was the variable that improved function and rotator cuff healing remains unclear. Recently, several randomized controlled trials have been published on this topic [10, 26, 29], affording the opportunity to perform a new meta-analysis to help resolve this controversy.

The benefit of our meta-analysis is that we pooled the data to more powerfully estimate the effect of PRP in arthroscopic rotator cuff repair. We pooled 13 randomized controlled trials, showing that PRP decreases retear rates and most clinical outcomes, including constant shoulder scores, constant pain scores, UCLA shoulder scores, and SST scores.

Chahal et al. [58] performed a meta-analysis including various study types, such as randomized controlled trials, cohort studies, and case-control trials, although only two randomized controlled trials were included. Another meta-analysis performed by Zhang et al. [18] omitted a high-quality randomized controlled trial [33] and included one nonrandomized controlled trial. In a meta-analysis including five studies performed by Cai et al. [56], only level I evidence studies were considered. This may have increased the likelihood of selection bias. Zhao et al. [19] included eight randomized controlled trials and concluded that PRP gives similar retear rates and clinical outcomes as the control repair method does. However, one study included in their data only reported on the retear rate at 3 months, which was distinct from that reported in other studies. Fu et al. [57] evaluated a total of 11 studies in a meta-analysis, eight of which included patients with full-thickness rotator cuff tears. Functional score data were included in the subgroup analyses. Overall, the standard difference in means of the functional scores was similar between patients who were administered PRP/fibrin matrix and patients in the control group. Warth et al. [17] included 11 studies in their meta-analysis and reported overall similar outcome scores and retear rates between patients who received PRP and those who did not. However, they found that when the initial tear size was greater than 3 cm in the anterior–posterior length, the PRP group had decreased retear rates after double-row repairs (25.9% vs. 57.1%; *P* = 0.046).

The present analysis included more randomized controlled trials using a more extensive and updated search. The enlarged sample size provides more accurate estimates of the effects of PRP on rotator cuff repair.

### Limitations

This meta-analysis has several limitations. First, this study possesses the potential for selection bias, performance bias, detection bias, attrition bias, and reporting bias, as is the case with any meta-analysis. Therefore, we conducted a thorough risk-of-bias assessment and presented the results in Fig. 2 to aid in data interpretation. Second, tear size may affect the differences between the two groups. No adequate studies report the outcomes of subgroups classified by tear size. Therefore, to ensure the rationality and validity of our meta-analysis, we did not perform subgroup analysis based on tear size. Third, some functional scores, such as the constant shoulder score, have not been specifically validated for use in rotator cuff outcome studies. However, the score has been widely used in the literature and may well be appropriate for the rotator cuff literature.

## Conclusions

Our systematic review and meta-analysis supports the use of PRP in the arthroscopic repair of rotator cuff tears. PRP may decrease retear rates and improve the clinical outcomes of arthroscopic rotator cuff repair.

## Data Availability

All data generated or analysed during this study are included in this published article.

## Abbreviations

ASES: American Shoulder and Elbow Surgeons
CIs: Confidence intervals
PRP: Platelet-rich plasma
RCTs: Randomized controlled trials
SST: Simple shoulder test
UCLA: University of California at Los Angeles
VAS: Visual analogue scale
